# Health literacy in Saudi Arabia: Implications for public health and healthcare access

**DOI:** 10.1002/prp2.514

**Published:** 2019-08-08

**Authors:** Rasha Almubark, Mada Basyouni, Ashjan Alghanem, Nora Althumairi, Dalal Alkhamis, Lamya S. Alharbi, Nouf Alammari, Aljoharah Algabbani, Fatemah Alnofal, Amani Alqahtani, Nasser BinDhim

**Affiliations:** ^1^ Saudi Food and Drug Authority Riyadh Saudi Arabia; ^2^ Ministry of Health Riyadh Saudi Arabia; ^3^ King Abdulaziz Medical City Riyadh Saudi Arabia

**Keywords:** health literacy, population health, Saudi Arabia

## Abstract

This study aims to describe the distribution of low health literacy (HL) in the population in the Kingdom of Saudi Arabia (KSA), and to analyze factors associated with low HL in KSA. A cross‐sectional national survey using quota sampling, population‐based of residents of KSA conducted via phone interviews supplemented by in‐person interviews. The survey included an overall evidence‐based measurement of HL. Both descriptive statistics of the sample and a multivariable logistic regression model predicting low HL were developed. A total of 3557 surveys were available for analysis, and 46% of the respondents were classified as having low HL. In regression modelling, low HL was associated with older age groups (age 47‐56 odds ratio [OR] 1.60, 95% confidence interval [CI] 1.30‐1.97; age 57‐66 OR 1.38, 95% CI 0.98‐1.94), the regions of Ha’il (OR 0.65, 95% CI 0.5‐0.85) and Najran (OR 1.27, 95% CI 0.99‐1.64), having been formerly married, lower levels of education (less than elementary OR 3.20, 95% CI 2.10‐4.88; and elementary, OR 1.62, 95% CI 1.14‐2.30), lower levels of income, and having sought healthcare exactly three times in the last year. Approximately half of KSA has low HL, and risk factors for low HL were older ages, lower income and education, having been formerly married, and a moderate pattern of health use. Future studies are needed to better characterize the distribution and determinants of low HL across KSA.

**Abbreviations**
ED: emergency department
GHL: general health literacy
HL: health literacy
KSA: Kingdom of Saudi Arabia
MoH: Ministry of Health

## 1 INTRODUCTION

Health literacy (HL) has been defined as occurring “when health information and services created for the public match with people’s capacity to find, understand, and use them.”^1^ HL refers to many types of literacy about health‐related topics, including medication use,^2^ understanding the appropriateness of emergency department (ED) use,^3^ understanding instructions with respect to oral care,^4^ and medical decision‐making and advice‐seeking.^5^

Having a high level of HL in a population is important for a number of reasons. First, those with low HL ultimately receive poorer care and are consequentially in poorer health. Illustratively, a high‐quality study was designed aimed at improving the medication literacy of patients being discharged from cardiac care for the purpose of preventing hospital readmission due to inappropriate medication use.^6^ Second, those with low HL are more likely to overuse the healthcare system, as well as drive up healthcare costs.^7^, ^8^

For these reasons, it is important to measure the levels of HL in populations in order to craft efforts to increase them. One population study in Taiwan benchmarked the Taiwanese level of HL at 34.4 on the general health literacy (GHL).^9^ In a study on Iraqis, the prevalence of inadequate HL was estimated at 30.3%.^10^ Studies in other populations have suggested that higher prevalence of low HL can complicate the delivery of healthcare at the population level,^11^ so tracking HL in the population can become important.

The Kingdom of Saudi Arabia (KSA) continues to expand and improve its extensive healthcare system.^12^ Under the current country‐wide strategic plan, Vision 2030, adjunctive independent businesses are encouraged to spring up to support core governmental infrastructures,^13^ and this will undoubtedly expand healthcare and increase access to cutting‐edge technological treatments. The healthcare system in KSA is divided into a public government sector and a private sector.^12^ The government side, both the Ministry of Health (MoH) as well as other government agencies administrate public healthcare facilities and delivery.^12^ Specifically, the military health system (including the National Guard Health Affairs) and other specialty healthcare settings such as Arabian American Oil Company (ARAMCO) Health Services are run under agencies other than the MoH, while the MoH is in charge of the network of community Primary Health Care Clinics (PHCCs) as well as larger public hospitals.^12^ Non‐Saudis living in Saudi Arabia have access to the healthcare system; they may maintain private insurance to access private facilities, or access the public facilities free‐of‐charge.

Unfortunately, healthcare services are concentrated in urban centers, which poses a challenge for connecting access to rural areas.^12^ Private health services are expanding, and are envisioned under Vision 2030 to support the public infrastructure.^12^, ^13^ Under Vision 2030, the strategy will be to improve and expand healthcare to meet the needs of the population, while incorporating features and services from the private sector strategically, such as private sector health insurance.^14^ While the healthcare system is well‐developed and represents a benefit to the population, those with low HL stand to be at higher risk for lack of access to healthcare and healthcare‐associated morbidity and mortality. While this is an overall advantage to the population, those with low HL stand to be at higher risk for healthcare‐associated morbidity and mortality. Therefore, it is now becoming increasingly more important to benchmark and track low HL in KSA at a population level.

Because KSA is a high income country,^12^ its citizens have benefitted from a high quality‐of‐life. Residents of KSA have adequate incomes,^12^ and while the higher education infrastructure within KSA is expanding, as part of a Vision 2030 push toward higher education, many students have obtained their post‐secondary education at venerable institutions outside KSA.^15^

However, it is not clear if higher education and income levels in KSA will necessarily protect its population from the negative risks conferred by low HL. One survey of type II diabetes patients in Riyadh showed that their main sources of health information were their physician, the television and the internet.^16^ Another study of patients with asthma in KSA found that approximately one third relied on the internet for health information about asthma.^17^ While the internet can be the source of correct health information, studies have shown that patient experiences are not ideal and often the information being sought is not actually found.^18^

In addition, low HL can be associated with negative medication‐related events, such as adverse drug events (ADE) and adverse drug reactions (ADR).^19^ Because studies in the KSA have found high rates of chronic disease in the population,^20^, ^21^, ^22^ it is possible that low levels of HL could present a challenge in KSA.

No previous studies have identified health literacy at national level in KSA. This study aims to describe the distribution of low HL in the KSA population, and to analyze factors associated with low HL in KSA.

## 2 MATERIALS AND METHODS

### 2.1 Study design

This was a nationwide cross‐sectional survey conducted in Saudi Arabia from February to August 2018. The study was conducted using a web‐based computer‐assisted phone interview. The study was reviewed and approved by the Saudi Food and Drug Authority (SFDA) Ethics Committee (NCBE # H‐01‐R‐070).

### 2.2 Participants and setting

The participants were included in the study if they met the following criteria: (a) they were a resident of Saudi Arabia (including both Saudis and non‐Saudis), (b) they were aged ≥18 years at time of survey, and (c) they were fluent in spoken Arabic. Participants were contacted by phone or in person, and were first asked demographic questions (including region of residence) for the variables included in the weighting strategy. Participants were excluded if quotas for cell in the weighting strategy in which the participant fell were full.

### 2.3 Sampling

This study used quota sampling to obtain a representative sample of respondents across the 13 regions of KSA stratified by gender and age. For the purposes of sampling, age was stratified into two groups based on the KSA median age, which was 37. After also stratifying by gender (male and female), there was a total of 52 quotas.

### 2.4 Sample size

Determination of sample size was based on the ability to compare age and gender groups between regions with medium effect size around 0.3. Thus, in each quota the sample size was 68 participants. The total target sample was n = 3536 participants representing all 13 regions. Once the quota sample is reached, the participants with similar characteristics will not be eligible to participate in the study.

### 2.5 Data collection approach

A random phone number list was generated from a governmental database and Sharik database. Sharik database is a database of individuals who are interested in participation in health research.^23^ This database was created in early 2018 by gathering phone numbers from two differnt minstries in KSA: The Ministry of Hajj and Umra (MoHU) and the MoH. The MoHU had provided contact information for pilgrims that had been collected, and the MoH provided contact information for individuals who had called into their “937” call center, which is a national phone number for information from the MoH. The call center began managing the data in the Sharik database, calling numbers and grooming the database to improve its accuracy. Ultimately, the sampling represented convenience sampling, but we made efforts to stratify to obtain as representative of a population as possible. However, it is true that bias will be present due to the nature of how we obtained the original set of phone numbers. Potential phone respondents were contacted by phone on up to three occasions. The questionnaire was administered by phone and data were recorded in web‐based software (described below).

Due to lack of phone numbers available, approximately 10% of the sample was interviewed in person during the data collection period. Those contacted for in‐person interviews were recruited from public spaces such as parks and shopping malls. Most of the population available in these places included women of all ages, some with children, as well as doing activities like shopping. Both phone and in‐person interview time was approximately 10 minutes.

The data from both phone and in‐person interviews were recorded via a web‐based software that was developed for this study called QPlatform®.^24^ This software integrates the eligibility and sampling modules including three questions for determining eligibility for sampling strata. Once these questions are answered, the software prevents ineligible respondents from continuing with the interview. In this study, and in order to submit a surevy, QPlatform® was programmed to require an answer to all survey questions (except for the questions of skip patterns).

### 2.6 Survey development

After providing verbal consent, the questions in the survey were about the following topics: demographics, clinical characteristics, and health literacy levels. Overall health literacy was measured using the question, “How often do you have someone help you read hospital materials?” with the following answers offered: never, rarely, sometimes, always, don’t know/refused. Both “rarely” and “never” were classified as having adequate HL, and all other answers were considered low HL.^10^ This question was based on a question from the Single‐Item Literacy Screener (SILS).^10^ This instrument has been translated into Arabic and studied for its validity and reliability.^10^ More than one HL question was not included because the questionnaire was already very long.

### 2.7 Measurements

Gender was estimated by the data collection interviewer. Gender and age were both used in representative sampling, and considered as potential confounders in the regression analysis. Region of residence of the respondent in KSA was collected also as part of sampling, and considered a potential confounder in the regression analysis, as studies have shown that there are both individual and community determinants of HL.^25^ Nationality (Saudi vs non‐Saudi) also was collected as a potential confounder based on previous studies.^26^ Smoking and marital status were measured as they have been used in previous HL prediction models.^25^, ^27^ Smoking status was classified as “yes, daily”, “yes, but not daily”, “no”; the top two levels were collapsed into a “yes” variable for regression modeling. Marital status was classified as “never married”, “married”, “widowed”, “divorced”, “separated but not divorced”, and don’t know/refused (DK). The categories of widowed, divorced, and separated but not divorced were combined into one variable for regression modeling.

Highest level of education question was considered a potential confounder, because education level has been found to be positively correlated with HL.^28^ The question was classified by falling in one of the following categories: less than elementary, elementary, secondary, graduated high school, diploma, bachelors, masters, doctorate, or DK. Answers of less than elementary, elementary, and secondary indicate the individual did not graduate high school. Diploma indicates a two‐year college degree, a bachelors a 4‐year college degree, a masters a two‐year post‐graduate degree, and a doctorate is a degree obtained post‐masters. For regression modeling, bachelors, masters, and doctorate were combined as the reference group.

Employment status was classified as employed, seeking employment, not employed, student, self‐employed, retired, unable to work, other, and DK. Employment status has been measured in studies of HL as a potential confounder.^29^

Health information‐seeking has been found to be positively correlated with household income in studies both outside^30^ and inside KSA,^16^, ^17^ which suggests that household income is also associated with HL levels. For these reasons, total household income in the previous month was collected as a potential confounder. Respondents were asked to classify their total household income in the previous month in the following categories: <5000 SAR (<$1300), 5000 to <10 000 SAR ($1300 to $2600), 10 000 to <15 000 SAR ($2600 to $4000), or 15 000 SAR or more ($4000 or more). We also measured healthcare use in the last 12 months with the question, “In the past 12 months, not counting times you went to an emergency room, how many times did you go to a doctor, nurse, or other health professional to get care for yourself?”. The answers offered were 0, 1, 2, 3, 4, or 5 or more times (or DK). We measured this as a potential confounder because high healthcare use has been found to be associated with low HL.^3^, ^31^

Overall health literacy was measured using the question, “How often do you have someone help you read hospital materials?” with the following answers offered: never, rarely, sometimes, always, don’t know/refused. Both “rarely” and “never” are classified as having adequate HL, and all other answers are considered inadequate or low HL.^10^ This question is based on a question from the Single‐Item Literacy Screener (SILS).^10^ This instrument has been translated into Arabic and studied for its validity and reliability.^10^

### 2.8 Data analysis

R software was used for analysis.^32^ First, a descriptive analysis of demographic and clinical characteristics was developed for the respondents in the sample. Next, a best‐fitting multivariable logistic regression model was developed using a stepwise selection approach.^33^ Criteria for covariates being retained in the model after each iteration included either having a coefficient with a *P*‐value of less than 0.05, or having a *P*‐value close to this number but being considered an important covariate to be retained in the model for empirical reasons. Data were weighted based on the 2017 Saudi census data to account for the probability of selection in each of the strata defined by age, gender, and region.

## 3 RESULTS

### 3.1 Characteristics of the participants

A total of 3557 participants completed the survey. Their mean age was 36.9 years (SD ± 11.19) and 51% of them were male. Forty‐seven percent of participants had a Bachelor degree level or higher qualification and 53% were employed. Majority of participants (80%) reported that they had overall excellent to very good health status. Less than a quarter (23%) reported having one or more chronic medical conditions. More details of participants’ socio‐demographic characteristics are presented in Table 1.

**Table 1:** Socio‐demographic characteristics of the participants (N = 3557)

Socio‐demographic characteristics	Mean	SD
Age (y)	36.9	± 11.36
	**N (%)**	**Weighted %**
Gender		
Female	1811 (50.91)	61.00
Male	1746 (49.09)	38.99
Education level		
Have not completed elementary school	141 (3.96)	4.50
Completed through elementary school	152 (4.27)	4.36
Completed through secondary school	239 (6.72)	8.31
Graduated high school	974 (27.38)	28.23
Diploma	362 (10.18)	10.93
Bachelor’s degree	1511 (42.48)	37.99
Master’s degree	126 (3.54)	4.61
Doctorate degree	29 (0.82)	0.75
Employment		
Employed	1893 (53.22)	56.75
Seeking employment	172 (4.84)	4.17
Not employed	669 (18.81)	17.33
Student	301 (8.46)	5.66
Self‐employed	160 (4.50)	5.43
Retired	196 (5.51)	7.18
Unable to work	55 (1.55)	1.27
Nationality		
Saudi	3087 (86.97)	81.62
Non‐Saudi	462 (12.99)	17.98
Marital status		
Never married	804 (22.60)	18.21
Currently married	2540 (71.41)	76.02
Widowed	89 (2.50)	2.56
Divorced	87 (2.45)	2.36
Separated but not divorced	18 (0.51)	0.40
Monthly income		
<5000 SR	705 (19.82)	20.56
5000 SR to < 10 000	861 (24.21)	25.42
10 000 SR to < 15 000 SR	683 (19.20)	18.30
≥15 000 SR	732 (20.58)	21.77
Health status		
Excellent	1794 (50.44)	54.42
Very good	1080 (30.36)	28.48
Good	543 (15.27)	13.88
Fair	97 (2.73)	2.02
Poor	35 (0.98)	1.01
Regions		
Aljouf	259 (7.28)	1.37
Northern Borders	264 (7.42)	1.00
Tabuk	267 (7.51)	2.52
Hail	271 (7.62)	2.05
Almadinah	273 (7.68)	6.25
Alqasim	277 (7.79)	4.33
Makkah	275 (7.73)	26.42
Al Riyadh	292 (8.21)	26.58
Eastern Province	281 (7.90)	15.53
Al Bahah	278 (7.82)	1.48
Asir	269 (7.56)	6.30
Jizan	271 (7.62)	4.43
Najran	280 (7.87)	1.67
Cigarette smoking		
Yes, daily smoker	494 (13.89)	17.15
Yes, but not daily	200 (5.62)	6.48
Nonsmoker	2863 (80.49)	76.35
Chronic conditions		
Diabetes	364 (10.23)	13.79
High blood pressure	304 (8.55)	10.69
Heart disease	79 (2.22)	2.66
Lung disease	100 (2.81)	2.31
Arthritis and rheumatism	159 (4.47)	4.43
Depression and anxiety	51 (1.43)	1.31
Other chronic diseases	199 (5.59)	6.33
Health literacy: how often respondent has help reading hospital materials		
Never	1411 (39.67)	40.96
Rarely	525 (14.76)	13.04
Sometimes	1134 (31.88)	31.65
Always	467 (13.13)	13.88

A total of 3557 surveys were available for analysis. As shown in Table 1, 46% of the respondents were classified as having low HL (see Table 1).

Overall, 46% (n = 1621) of the sample was classified as having low HL (Weighted percentage = 45.99%, adjusted by Age, gender and region), and women made up a slightly larger percentage of those with low HL compared to men (51% vs 49%). Table 2 shows that those in older age groups were overrepresented among those with low HL (ages 47‐56 made up 17% of low HL vs 10% of not low HL; ages 57‐66 made up 6% of low HL vs 4% of not low HL; and ages 67 and older made up 2% of low HL vs 1% of not low HL). In terms of region, all regions were roughly equally represented in the low HL group compared to the not low HL group except Ha’il, which was underrepresented among those with low HL (6% vs 9% of those without low HL), and Najran, which was overrepresented among those with low HL (9% vs 7% of those without low HL).

**Table 2:** Characteristics of the participants by health literacy status

Characteristics	Health literacy status (n, %)		*P*‐value
	Low health literacy (1621, 46%)	Not low health literacy (1936, 54%)	
Gender			
Male	791, 49%	1020, 53%	0.0228
Female	830, 51%	916, 47%	
Age group			
18‐36	755, 47%	1056, 54%	<0.0001
37‐56	743, 46%	797, 41%	
57+	123, 8%	83, 5%	
Region			
Al Jouf	127, 8%	132, 7%	0.0114
Northern Borders	116, 7%	148, 8%	
Tabuk	114, 7%	153, 8%	
Ha’il	94, 6%	177, 9%	
Al Madinah	133, 8%	140, 7%	
Al Qasim	122, 8%	155, 8%	
Makkah	126, 8%	149, 8%	
Al Riyadh	134, 8%	158, 8%	
Eastern Province	130, 8%	151, 8%	
Al Bahah	136, 8%	142, 7%	
Asir	125, 8%	144, 7%	
Jizan	115, 7%	156, 8%	
Najran	149, 9%	131, 7%	
Nationality			
Saudi	1408, 87%	1679, 87%	0.9788^a^
Non‐Saudi	209, 13%	253, 13%	
Marital status			
Never married	322, 20%	482, 25%	<0.0001
Currently married	1164, 72%	1376, 71%	
Widowed	62, 4%	27, 1%	
Separated or divorced	59, 4%	46, 3%	
Less than high school	316, 20%	216, 11%	
Graduated High School	405, 25%	569, 29%	
Diploma or college degree	820, 50%	1053, 54%	
Higher education	61, 4%	94 ,5%	
Employment status			
Employed	828, 51%	1065, 55%	<0.0001
Not employed/seeking employment	396, 24%	445, 23%	
Retired or unable to work	137, 8%	114, 6%	
Student	115, 7%	186, 10%	
Household income			
<5000 SR	363, 22%	342, 18%	<0.0001
5000 to < 10 000 SR	399, 25%	462, 24%	
10 000 to < 15 000 SR	311, 19%	372, 19%	
15 000 SR or more	331, 20%	401, 21%	

All percentages are column percentages except in this row; row percentages are presented to show the distribution of characteristics across health literacy status. NA, Not Applicable.

a Fisher’s exact test.

As shown in Table 2, a trend was observed in that those with any college education (diploma or above) were overrepresented among those with adequate HL, and those with the highest level of education being less than elementary, elementary, or secondary school making up 20% of those with low HL compared to 11% of those with not low HL. In terms of income, there was a trend toward higher income being associated with higher HL. More details are presented in Table 2.

### 3.2 Clinical characteristics

As shown in Table 3, smoking status was not associated with HL. Half of those surveyed reported excellent health (50%), and another 30% reported very good health. Among respondents, about one quarter (24%) did not use healthcare within the last 12 months.

**Table 3:** Clinical characteristics of sample by health literacy status

Characteristics	Health literacy status n, %		*P*‐value
	Low Health Literacy (1621, 46%)	Not Low Health Literacy (1936, 54%)	
Cigarette smoking status			
Yes, daily	210, 13%	284, 15%	0.0527
Yes, but not daily	79, 5%	121, 6%	
No	1332, 82%	1531, 79%	
General health status			
Excellent	775, 48%	1019, 53%	0.0003
Very good	485, 30%	595, 31%	
Good	278, 17%	265, 14%	
Fair	58, 4%	39, 2%	
Poor	19, 1%	16, 1%	
Number of times sought healthcare in past 12 months			
None	353, 22%	490, 25%	<0.0001
Less than five times	830, 51%	1012, 52%	
Five times or more	417, 26%	403, 21%	
Chronic medical conditions			
Diabetes	196, 12%	168, 9%	0.0010
Hypertension	173, 11%	131, 7%	<0.0001
Heart condition	47, 3%	32, 2%	0.0165
Respiratory condition	50, 3%	50, 3%	0.4237
Arthritis	89, 5%	70, 4%	0.0090
Depression/anxiety	29, 2%	22, 1%	0.1365
Other chronic condition	108, 7%	91, 5%	0.0138
Did not report any chronic conditions asked about	1184, 73%	1548, 80%	<0.0001

All percentages are column percentages except in this row; row percentages are presented to show the distribution of characteristics across health literacy status. NC, Noncalculable.

In terms of chronic conditions, over three‐fourths of the respondents (77%) did not report any chronic conditions. The most commonly reported chronic conditions were diabetes (10%) and hypertension (9%). Diabetics and hypertensives were overrepresented among those with low HL (12% vs 9% among not low HL for diabetics, and 11% vs 7% among not low HL for hypertensives).

### 3.3 Factors associated with low HL in KSA

As shown in Table 4, the only age groups that were found to have statistically significant association with low HL were those aged 47‐56, who had 60% odds of low HL compared to the comparison age groups (95% CI 1.30 to 1.97).

**Table 4:** Factors associated with low HL in KSA

Category	Level	Outcome: low health literacy	
		Odds ratio	95% confidence interval
Age group			
18‐26		Reference	Reference
27‐36		Reference	Reference
37‐46		Reference	Reference
47‐56		1.60	1.30‐1.97
57‐66		1.38	0.98‐1.94
67+		Reference	Reference
Region			
Ha’il		0.65	0.50‐0.85
Najran		1.27	0.99‐1.64
All other provinces		Reference	Reference
Marital Status			
Never married		Reference	Reference
Married		Reference	Reference
Widowed, divorced, or separated but not divorced		1.41	1.03‐1.94
Don’t know/refused		Reference	Reference
Highest level of education			
Less than elementary		3.20	2.10‐4.88
Elementary		1.62	1.14‐2.3
Secondary		Reference	Reference
Graduated HS		Reference	Reference
Diploma		Reference	Reference
Bachelors		Reference	Reference
Masters		Reference	Reference
Doctorate		Reference	Reference
Unknown		Reference	Reference
Household income			
<5000 SR		1.32	1.09‐1.61
5000 to < 10 000 SR		1.25	1.04‐1.49
10 000 to < 15 000 SR		1.23	1.02‐1.49
15 000 SR or more		Reference	Reference
Don’t know/refused		Reference	Reference
Number of times sought healthcare in past 12 months			
None		Reference	Reference
One time		Reference	Reference
Two times		Reference	Reference
Three times		1.44	1.15‐1.79
Four times		Reference	Reference
Five or more times		Reference	Reference
DK/refused		Reference	Reference

Levels of categorical variables were modeled as indicator variables, with the following reference groups designated: age 18‐26; Al Riyadh; married; education at the levels of Bachelors, Masters, or Doctorate; household income 15 000 SR or more; and seeking healthcare zero times in the past 12 months. Indicator variables that did not meet the criteria to be retained in the model were removed, and whenever that happened, that level joined the reference group.

In terms of region, the only region that had statistically significant association between low HL compared to the comparison regions was Ha’il, which had only 65% the odds of the comparison provinces of having low HL (95% CI 0.50‐0.85). Najran was associated with 27% odds associated with low HL compared to the comparison provinces (95% CI 0.99‐1.64), but this comparison also did not rise to the level of statistical significance.

With respect to marital status, compared to individuals who have never been married or are currently married, those who were formerly married (widowed or divorced) or who are separated but not divorced had over 40% odds associated with low HL (95% CI 1.03‐1.94), and this relationship was statistically significant. For highest level of education, those with less than elementary education had an association of over three times the odds of low HL (odds ratio [OR] 3.20, 95% CI 2.10‐4.88), and those with only elementary education had an association of more than 60% odds of low HL (1.62, 95% CI 1.14‐2.30) compared to the other educational group levels, and these relationships were statistically significant. Compared to the highest level of monthly household income (and those who did not provide an answer), the first three income levels all were statistically significantly associated with a quarter to a third odds of low HL (<5000 SR 1.32, 95% CI 1.09‐1.61; 5000 to <10 000 SR, 95% 1.25, 95% CI 1.04‐1.49; 10 000 to <15 000 SR, 1.23, 95% CI 1.02‐1.49). Hence, there was no dose‐response relationship with regard to the association between income levels and HL in KSA.

Finally, in terms of number of times the respondent sought healthcare in the last 12 months, those reporting visiting healthcare three times had 44% statistically significant association of low HL (95% CI 1.15‐1.79) compared to those reporting zero times, one time, two times and four or more times.

## 4 DISCUSSION

This analysis suggests that almost half of the residents of KSA had low HL. In descriptive analysis, women and those who were not employed were overrepresented among those with low HL. However, these relationships disappeared in multivariable analysis. Final regression results suggest that older age groups, those from the Najran region, the formerly married, those with less than a high school education, those in lower income groups, and moderate users of healthcare were at higher risk for low HL. In addition, those in the region of Ha’il were at substantially lower risk of low HL.

The estimate of 46% of our representative sample having low HL is high but not inconsistent with other estimates from the literature. Earlier, it was mentioned that an Iraqi study showed a prevalence of inadequate HL of 30.3%.^10^ This is a particularly useful comparison figure for this study, as these authors used the same Arabic‐worded question and classification algorithm for low HL as was used in this study.^10^

Our analysis showed significant independent relationships between certain older age groups, having been formerly married, lower highest education level, lower income level, and moderate healthcare use and low HL. These risk factors for low HL are consistent with the literature and were deliberately included in multivariable modeling for this reason.^25^, ^28^ However, it is important to note that indicator variables from only two of five levels of age, two levels of education, one level of marital status, and mysteriously, one level of healthcare use (three times in the last year) survived in the multivariable model. Because selective stepwise modeling was used to generate the model, the authors believe that this is evidence of collinearity between these variables, although there may be reasons other than collinearity, and further research is needed to better characterize their independent contributions to low HL in KSA.

In terms of the finding regarding increased risk of low HL associated with exactly three healthcare visits in the last year (rather than two, or four of more), one is forced to suspect either an artifact in the form of a spurious association, or else, an underlying periodicity in the data that has not yet been identified. What would have been expected was a dose‐response relationship representing that more visits would be associated with higher risk of low HL, but in our data, this was not the case. In a study using United States (US) Veteran’s Health Administration (VHA) data which included scores on HL assessments, it was found that having inadequate or marginal HL was associated with an average higher per patient cost of about $30 000.^7^ This extra cost was born in a system that provides care free‐of‐charge to veterans, meaning that it represents a measurement of increased healthcare use. Another study found an association between low HL and higher rates of emergency department (ED) use.^3^

This finding of increased healthcare use and cost being associated with lower HL has implications for the goals of Vision 2030. In order to optimize healthcare utilization in KSA’s expanding healthcare system, the public will need to have the capacity to identify appropriate healthcare services and information, understand them, and use them effectively to improve their health. We estimated that approximately half of the public does not possess HL levels that are adequate to have the capacity to optimally utilize healthcare. Without remedying this, KSA could be inadvertently creating issues in healthcare and public health that it is actually seeking to solve.

Although this study did not reveal much in the way of regional variations in HL, it is important to highlight the finding that residents of Ha’il experienced a much lower risk of low HL compared to all the other provinces except Najran, which experienced statistically significantly higher odds of low HL compared to all other provinces. Both these findings do not have a ready explanation. The region of Ha’il has not been studied extensively, but residents have been found to have high levels of diabetes.^34^

It is also unclear why Najran residents were at higher risk for low HL, but this may have to do with relative lack of healthcare access in this region. KSA’s healthcare system is currently designed around large medical cities within urban areas, and efforts are being made to decentralize services and provide more primary health care centers (PHCCs) accessible to nonurban populations.^12^ In a survey in which 52.93% of respondents were from the Asir region (which abuts Najran on the west side), 43.23% of respondents reported taking medication. However, over half reported that they had inadequate information about side effects of their medication, potential adverse events from their medication, and symptoms of overdose from their medication.^35^ This lack of knowledge may be the result of living in an area with less healthcare access.

Taken together, these findings suggest that the subsequent steps with respect to addressing low HL in KSA should aim to empower those aged 40 and older with knowledge about medications, as well as the proper use of it, especially for common chronic diseases in KSA such as hypertension and type II diabetes. In addition, future research is needed to better understand regional variations in HL, and how healthcare access may mutually influence HL. The HL question used in this study referred to “hospital papers”—this suggests that more research should be done on how to present information optimally to patients in terms of clarity of wording, language (English, Arabic, or another), reading level, text formatting, and method—such as whether a nurse delivers the information or a pharmacist. Efforts to improve these features could impact how questions like the ones used in this study are answered by the population.

Our study is not without limitations. In our study, we used one database as our main phone list for contact, and only after we began the study did we realize that many of the numbers were out‐of‐date. This forced us to develop a second source of participants in order to fill quota cells to achieve a representative sample. Unfortunately, this may have introduced bias into the study. Specifically, the original members of the database may have been skewed toward higher education and socio‐economic level, as they either participated in Hajj or Umra, or called into the call center. These are both activities that would be easier to accomplish at a higher socio‐economic level, and represent typical patterns of behavior among those with higher education. Since HL is associated with educational level, this may have introduced bias. Since we completed data collection on this study, work on database has started to refresh its contact list using purchased lists and other methods to improve data quality. Next, we only included one question specifically regarding HL instead of a full instrument (mainly because our questionnaire was already quite long). We realize that the word “hospital” in the question may limit the respondents’ context; not all adults go to the hospital regularly. Hence, we will consider changing the wording of this question on future surveys.

In conclusion, our study in KSA found a prevalence of low HL in the population of 46%, and risk factors for low HL were older ages, lower income and education, having been formerly married, and having a moderate pattern of healthcare use. Future efforts should focus on further characterizing the distribution and determinants of low HL across KSA, and developing interventions to empower our population with the knowledge and skills they require in order to optimally benefit from KSA’s healthcare system.

## DISCLOSURES

The authors declare that there is no conflict of interest.

## AUTHORS’ CONTRIBUTION

Rasha Almubark: Designing the study, supervising data collection, conducting data analysis and writing up the manuscript. Mada Basyouni: Designing data collection tool. Ashjan Alghanem: Designing data collection tool. Nora Althumairi: Data collection. Dalal Alkhamis: Data collection. Lamya S. Alharbi: Data collection. Nouf Alammari: Data collection. Aljoharah Algabbani: Data collection. Fatemah Al nofal: Data collection. Amani S. Alqahtani: Editing the manuscript. Nasser F.BinDhim: Designing the study, supervising study progress. All authors have made substantial contributions to editing the manuscript.

## Data Availability

The data that support the findings of this study are available on request from the corresponding author. The data are not publicly available due to privacy or ethical restrictions.
